# Prevalence and Associated Risk Factors of Gastrointestinal Parasitic Infections in Local and Cross-Border Cattle in Kanchanaburi Province, Thailand

**DOI:** 10.3390/vetsci13080746

**Published:** 2026-07-28

**Authors:** Khondoker Mohammad Iftekhar Zahan, Wissanuwat Chimnoi, Roshni Jahan Nur Antara, Chanya Kengradomkij, Narisorn Pilean, Ketsarin Kamyingkird, Tawin Inpankaew

**Affiliations:** 1 Department of Parasitology, Faculty of Veterinary Medicine, Kasetsart University, Lad Yao, Chatuchak, Bangkok 10900, Thailand; khondokermohammadiftekhar.z@ku.th (K.M.I.Z.); wissanuwat.c@ku.th (W.C.); roshnijahannur.a@ku.th (R.J.N.A.); fvetcyk@ku.ac.th (C.K.); ketsarin.ka@ku.th (K.K.); 2 Graduate Program in Animal Health and Biomedical Sciences, Faculty of Veterinary Medicine, Kasetsart University, Bangkok 10900, Thailand; 3 Department of Livestock Development, Sa Kaeo Animal Quarantine Station, Sa Kaeo 27180, Thailand; narisornpiper@gmail.com

**Keywords:** gastrointestinal parasites, cattle, prevalence, risk factors, Kanchanaburi province

## Abstract

Gastrointestinal (GI) parasitic infections are still a major concern for cattle health and productivity in tropical and subtropical regions. However, epidemiological data on GI parasitism in cattle from western Thailand remain limited, particularly in border areas with frequent animal movement. This study focuses on establishing the prevalence of gastrointestinal parasitic infections and identifies its related risk factors among the cattle in Kanchanaburi province, western Thailand. Cross-sectional study was performed from December 2022 to January 2023 involving 153 cattle from the following three study populations: local cattle from Thong Pha Phum district, Sangkhlaburi district, and cross-border cattle imported from Myanmar. Fecal samples were examined using both simple flotation and formalin-ethyl acetate sedimentation techniques, and data on animal and management factors were collected through questionnaire surveys. Overall, 35.9% (55/153) of the cattle had at least one GI parasite species positive. Six parasite taxa were identified, with Strongyle-type nematodes being the most frequently detected (26.1%), followed by other helminth parasites. The prevalence of infection and parasite diversity increased progressively in cattle populations located closer to the Thai–Myanmar border, suggesting that this region may represent a potential hotspot for GI parasite transmission. From a One Health perspective, these findings highlight the potential for some helminths to circulate across animal, human, and environmental interfaces. The presence of 1.3% for both *Capillaria* spp., *Trichuris* spp. and 0.7% of *Fasciola* spp. suggests possible zoonotic transmission pathways, particularly via the fecal-oral route. Although 93.5% of cattle had reportedly received anthelmintic treatment, no apparent protective effect was observed. These findings highlight that the Thai–Myanmar border acts as a hotspot for parasite transmission and the need for improved parasite control strategies in this region, alongside integrated One Health surveillance linking animal, human, and environmental health in this cross-border region.

## Simple Summary

Gastrointestinal (GI) parasites can negatively affect cattle health and productivity, especially in tropical regions. This study investigated GI parasitic infections in cattle from Kanchanaburi province, western Thailand. Overall GI parasite infection in cattle was 35.9%. Strongyle-type nematodes were the most common parasite found in this study. High GI parasite infection rates and parasite diversity were investigated in cattle in the Thai–Myanmar border. However, despite frequent anthelmintic use, its efficacy was never monitor, highlighting the need for better monitoring parasite control strategies.

## 1. Introduction

Gastrointestinal (GI) parasite infections are considered one of the most common health issues for cattle in the tropics and subtropics around the world. In tropic and subtropic areas, there are high temperatures and humidity, which promote the rapid transmission of helminths and protozoan parasites [1,2,3]. GI parasite infections result in huge economic losses through slower weight gain, reduced milk and meat production, lowered reproduction success, poor feed efficiency, reproductive inefficiency, and mortality in many severe cases [4]. Many abnormalities caused by nematodes, trematodes, and cestodes are economically important for livestock. Chronic infections often remain subclinical, but even minor illnesses can significantly diminish productivity and economic benefits for farmers [5]. The prevalent parasites shown in Thai cattle include Strongyle-type nematode genera, such as *Haemonchus*, *Cooperia*, *Ostertagia*, and *Trichostrongylus*, alongside *Paramphistomum* spp., *Moniezia* spp., *Capillaria* spp., *Trichuris* spp., *Fasciola* spp., and *Eimeria* spp. [4,5], the latter a coccidian protozoan reported with a pooled regional prevalence of 29.5% in Asia [6]. The prevalence of those parasites varies by area and management system. In a study conducted in Kalasin Province, Strongyle-type nematodes had the highest prevalence, at 84.24%, followed by *Fasciola* spp. and *Moniezia* spp. [4]. Also, in Nakhon Ratchasima Province, *Haemonchus contortus* and *Oesophagostomum radiatum* were identified as the dominant species in dairy and beef cattle, respectively [5].

Kanchanaburi province, located at the Thailand–Myanmar border region, harbors a considerable number of cattle from local farms and, through livestock commerce across the border, makes it a suitable site for the transmission of parasitic diseases [7]. There are some factors known to raise the risk of GI parasitic infection in this setting: reliance on natural river and pond water for drinking [8], seasonal variation in larval survival on pasture [9], inadequate fecal and pasture management [10], and the presence of other domestic and wild animals such as dogs, cats, and rats on the same farms [11]. Cats may serve as vectors, carriers, reservoirs, and definitive hosts for several GI parasites [12]. Similarly, birds and rodents increase the parasitic transmission [13]. Previous Thai surveys report considerable regional variation in GI parasite prevalence, from 45.3% in Nan Province [14] to over 60% in Sa Kaeo Province [7], yet data from Kanchanaburi Province itself remain limited despite its high cross-border cattle traffic and large number of small-scale farms.

Although GI parasite surveys have been conducted elsewhere in Thailand—in Kalasin [4], Sa Kaeo [7] Provinces, and Nan Province [14]—and an earlier survey covered local cattle within Kanchanaburi Province itself [15], none of these studies sampled cross-border cattle alongside local herds or examined risk factors for GI parasitism in this setting. The epidemiological pattern of GI parasitic infections in cattle in Kanchanaburi province, and specifically how it differs between resident and cross-border populations, therefore remains understudied. The diversity in the environment of this province, which is also a border province that uses natural water sources, makes it a possible hotspot for the spread of the parasites. Therefore, the purpose of this research is to identify the prevalence and its related risk factors of GI parasitic infections in cattle in Kanchanaburi province, Thailand.

## 2. Materials and Methods

### 2.1. Study Area and Sample Collection

**Figure 1:**
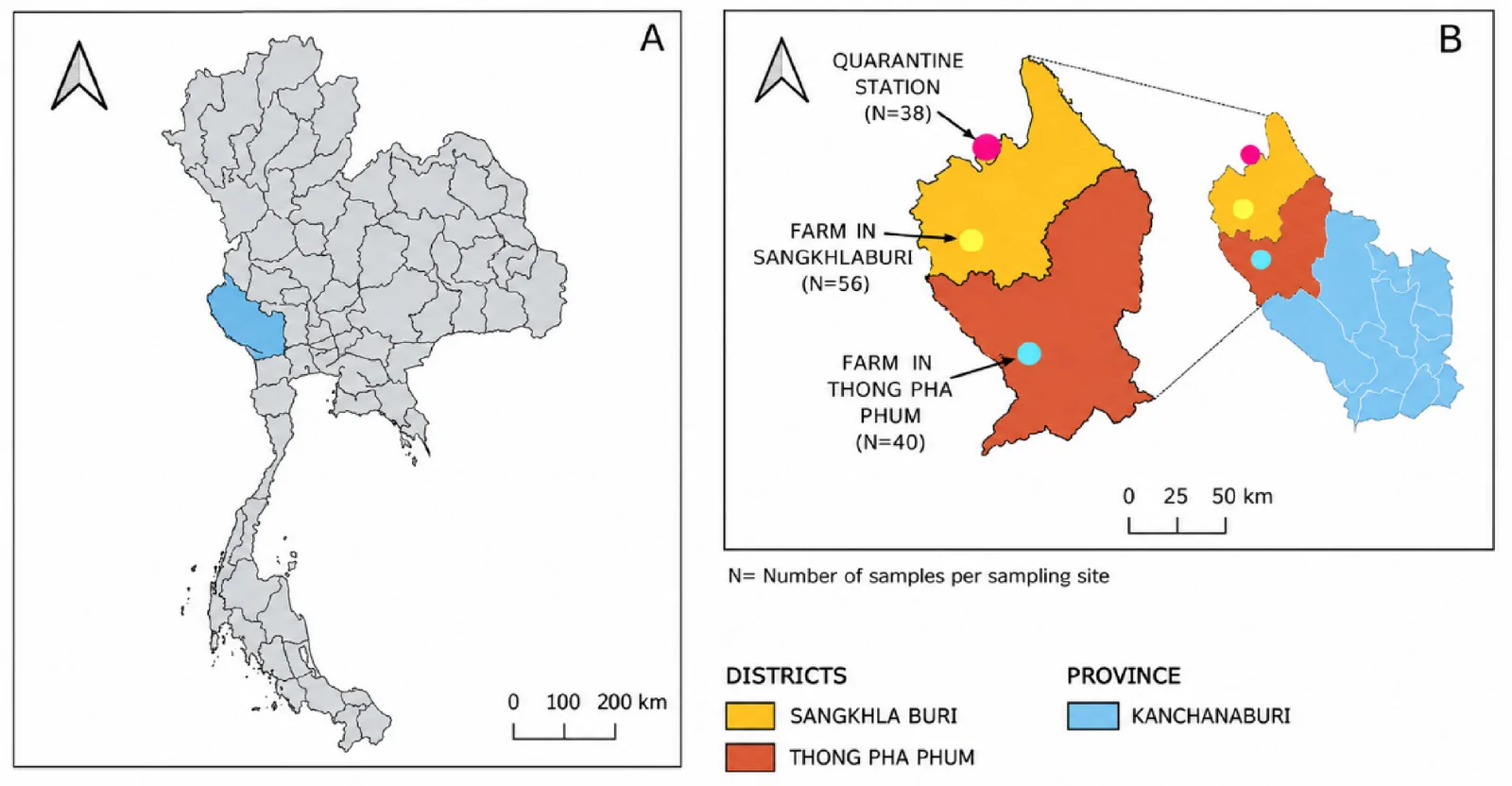
This geographic distribution of cattle farms in Kanchanaburi province, western Thailand. (**A**) Map of Thailand with province highlighted—the blue circle shows Kanchanaburi province. TH, Thailand. (**B**) Surveyed locations in Kanchanaburi.

The cross-sectional study was performed from December 2022 to January 2023 in Kanchanaburi province, located in western Thailand, adjacent to the Thai–Myanmar border. A total of 153 fecal samples were collected using a convenience-sampling approach, based on farm accessibility and owner consent rather than random selection, from cattle farms describing two districts, including 49 samples from Thong Pha Phum (14.840545° N, 98.492435° E) and 66 samples from Sangkhla Buri (15.150031° N, 98.450269° E), and from 38 cross-border cattle sampled at the Kanchanaburi quarantine station (15.256216° N, 98.419401° E; Sangkhla Buri) in Kanchanaburi, Thailand, drawn from the quarantine batches held at the station at the time of sampling. This area spans a total area of about 19,483 km^2^ and consists of hilly topography and major river systems such as Khwae Noi and Khwae Yai. The geographic sites of the sampling areas have been illustrated in Figure 1. The cross-border cattle group consisted of cattle originating from Myanmar that had entered Thailand, and they were held at quarantine stations for mandatory health inspection in accordance with the Thai Department of Livestock Development regulations.

The minimum required sample size was determined according to the formula developed by Thrusfield and Christley [16] for prevalence estimation studies:

*n* = *Z*^2^
*P_exp_*(1 − *P_exp_*)/*d*^2^

where *n* refers to the sample size required, Z represents to the desired confidence level (1.96 for 95% confidence), P_exp_ is the expected prevalence, and d indicates the desired absolute precision [16]. According to the previous study, the expected prevalence rates of bovine GI parasites in Thailand was assumed to be 35.7% [15] and adopting a 95% confidence level with 8% absolute precision, the computed minimum sample size was 138 animals. The final study sample (*n* = 153) therefore exceeded this threshold.

Each animal was humanely and appropriately restrained, and approximately 10–20 g of feces was collected from the rectum, where disposable gloves were used to prevent cross-contamination between animals and kept in labeled sterile plastic containers with the identification number, farm code, sampling district, and date of collection. Samples were stored in insulated cool boxes during transportation to the Parasitology Laboratory, Faculty of Veterinary Medicine, Kasetsart University, and also refrigerated at 4 °C until laboratory examination. Animal-level data, including age, sex, breed, pregnancy status, and abortion history, were recorded for each sampled animal. Animals were classified into the following two age groups: young (<1 year) and adult (≥1 year) [15]. In addition, farm-level management and environmental variables were collected as potential risk factors through structured questionnaires and direct on-site observations. These variables included age, sex, breed, pregnancy status, abortion history, water sources, feed storage type, presence of ectoparasites, presence of other domestic or wild animals (dogs, cats, chickens, wild birds, and rats), and finally, the deworming history (both anti-ectoparasites and anti-endoparasites). Presence of other domestic or wild animals on the farm, and whether dogs or cats were free-roaming (i.e., not confined or tethered and able to move beyond the immediate farm perimeter during the day) versus confined, were recorded based on the farmer’s questionnaire response describing routine farm conditions, cross-checked against direct observation at the time of the visit; these variables therefore reflect owner-reported routine status rather than a standardized behavioral assay.

### 2.2. Fecal Analysis

Fecal samples were analyzed for GI parasitic eggs using both the simple flotation [17] and the formalin-ethyl acetate sedimentation [18]. The flotation solution used was a saturated sodium chloride (NaCl) solution, prepared by dissolving around 360–380 g of NaCl in one liter of warm distilled water with continuous stirring until no further salt could dissolve. The solution was allowed to cool to room temperature, and its specific gravity was approximately 1.20, which was confirmed by a hydrometer. Approximately 2 g of feces was placed into a wide-mouthed disposable plastic cup, followed by the addition of 44 mL of the NaCl flotation solution and thorough mixing. An additional 4 mL of flotation solution was introduced and mixed. The fecal suspension was strained through a tea strainer into a clean receptacle. The filtrate was decanted into a 10–15 mL test tube positioned in a rack and topped with flotation solution until a convex meniscus formed at the tube’s aperture. A 22 × 22 mm coverslip was placed flush on the meniscus and left undisturbed for 10–15 min, after which it was carefully lifted and transferred to a labeled microscope slide for examination.

For the formalin-ethyl acetate sedimentation, 1–3 g of a homogenized cattle fecal sample. Then, 10% formalin was added to the sample, and the ratio was 1:10 (fecal sample: formalin). The sample was fixed in formalin solution at room temperature for at least 24 h. After fixation, the sample was centrifuged at 1500 rpm for 10 min. The supernatant was carefully decanted without disturbing the sediment, which was retained at the bottom of the tube. A similar volume of ethyl acetate was added to the sediment. The tube was shaken for 1–2 min to ensure thorough mixing and then allowed to stand at room temperature for 10–15 min. The mixture was centrifuged again at 1500 rpm for 10 min. The supernatant was carefully decanted, leaving the sediment at the bottom of the tube. The sediment was washed with distilled water or saline solution and centrifuged again at 1500 rpm for 10 min. The supernatant was then decanted. A clean glass slide was prepared for microscopic examination, and a drop of the concentrated sediment was placed onto the glass slide. The preparation was covered with a 22 × 22 mm coverslip for microscopic examination.

The parasites were identified based on their morphological features such as egg size, shape, and shell appearance, which were observed under light microscopy at 40× and 100× magnifications.

### 2.3. Statistical Analysis

Data were analyzed using Microsoft Excel 2024 and the R program version 4.05 [19]. Parasite prevalence for each taxon was expressed as the proportion of positive animals out of the total examined, with 95% confidence intervals (CIs). Associations between GI parasite positivity and potential risk factors were explored through univariable analysis applying the Chi-squared test or Fisher’s exact test (where expected cell counts were below 5) at a significance threshold of *p* < 0.05 with GI parasite status serving as the binary dependent variable and the animal and management factors instance of age, sex, breed, pregnancy status, abortion history, presence of ectoparasites, domestic or wild animals present in the farms (dogs, cats, chickens, wild birds, and rats), deworming history (both anti-ectoparasites and anti-endoparasites) as the independent variables.

## 3. Results

### 3.1. Study Population and Demographic Characteristics

Among 153 samples, 88 (57.5%) were female, and 65 (42.5%) were male. Almost all sampled cattle were adult (144/153; 94.1%), and young cattle were recorded only in Thong Pha Phum (6/49; 12.2%) and Sangkhla Buri (3/66; 4.5%). All 38 cattle from the Myanmar border site were male, consistent with livestock-trading and herding activities predominant in that area, whereas females made up the majority in Thong Pha Phum (73.5%) and Sangkhla (78.8%). Regarding breed, Mix AB or crossbred (*Bos indicus* × *Bos taurus*; locally referred to as Mix AB) animals predominated in Thong Pha Phum (81.6%), while local-breed cattle constituted the majority in Sangkhla Buri (72.7%) and represented all animals at the cross-border area (100.0%). Regarding pregnancy status, pregnant animals were mostly recorded in Thong Pha Phum (19/49; 38.8%) and less recorded in Sangkhla Buri (9/66; 13.6%); no pregnant animals were recorded among the cross-border cattle (0/38; 0.0%) (Table 1).

River water was the primary drinking source in Sangkhla Buri (77.3%) and the main source at the cross-border site of Thailand and Myanmar (100.0%), whereas piped water was the main source in Thong Pha Phum (75.5%). Internal antiparasitic treatment had been given to 143/153 animals (93.5%); the 10 untreated animals were all from Sangkhla Buri. Anti-ectoparasitic treatment was administered to 136/153 animals (88.9%), again with untreated animals concentrated in Sangkhla Buri (17/66; 25.8%). Ectoparasite infestation was recorded in 103 animals (67.3%) and was present only in Thong Pha Phum (95.9%) and Sangkhla Buri (84.8%); no ectoparasites were detected in the cross-border area. Dogs were present on 86.9%, and cats were present in Thong Pha Phum (75.5%) and Sangkhla Buri (62.1%) but were absent from the cross-border area. Wild birds were observed exclusively in Thong Pha Phum (100.0%), while cat roaming was observed only in Sangkhla Buri (12.1%). A history of abortion was recorded in only three animals overall (2.0%): two from Thong Pha Phum and one from Sangkhla Buri (Table 1).

### 3.2. Overall Prevalence of GI Parasites

**Figure 2:**
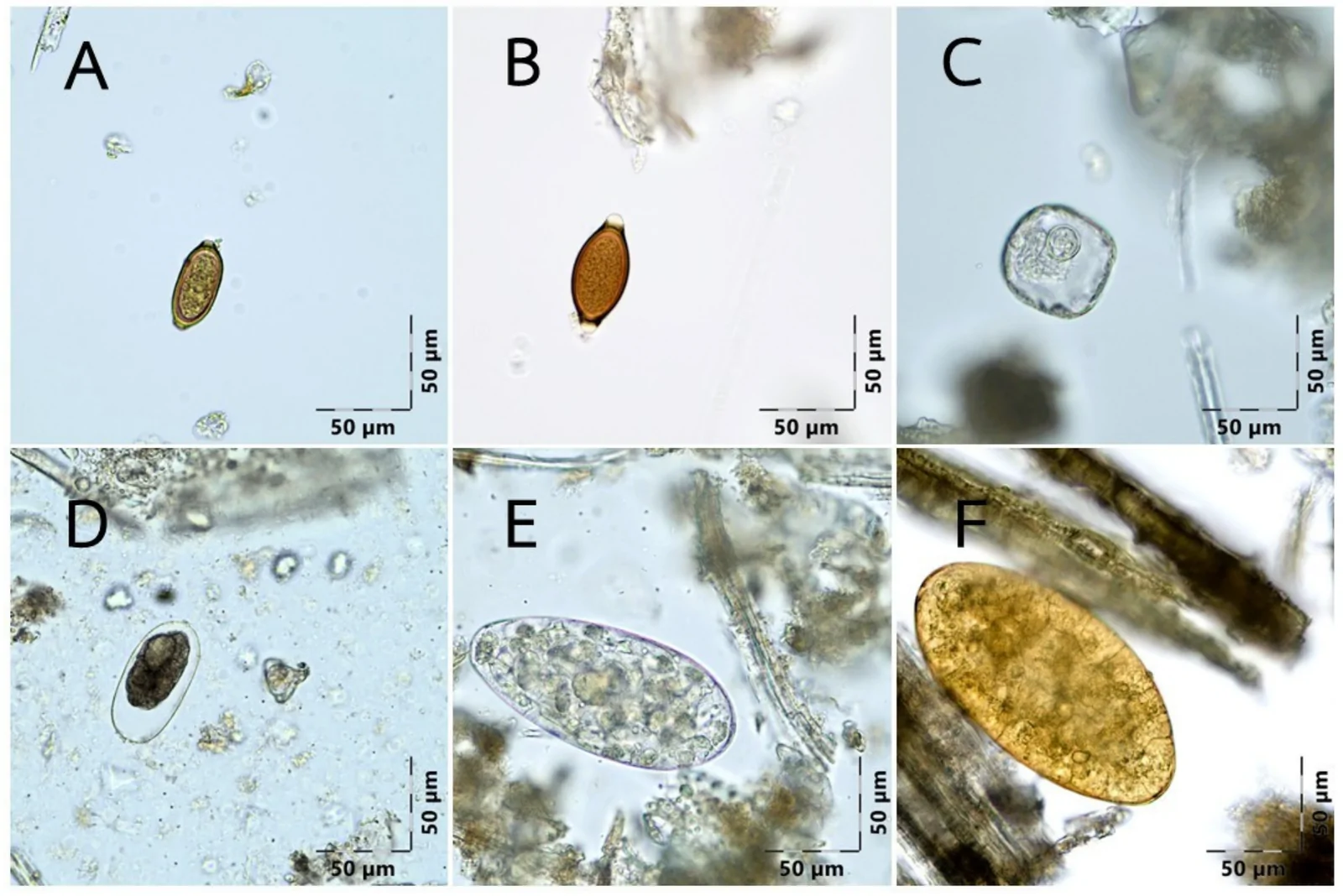
The morphological features of the diagnostic stages (eggs) of the GI nematodes parasitic to cattle based on coprological examination of the fecal samples of cattle. (**A**) *Capillaria* spp., (**B**) *Trichuris* spp., (**C**) *Moniezia* spp., (**D**) Strongyle-type nematode, (**E**) rumen fluke (*Paramphistomum* spp.), (**F**) liver fluke (*Fasciola* spp.). The fecal samples were viewed under 100× magnification after preparation and then measured to morphometric analysis. The scale bar represents 50 µm.

Of 153 cattle examined, 55 (35.9%) were positive for a minimum of a single GI parasite species. Six parasite species were identified across all study sites (Table 2). Strongyle-type nematodes were the most prevalent, detected in 40 animals (26.1%), followed by rumen fluke (*Paramphistomum* spp.) in 13 (8.5%), *Moniezia* spp. in three (2.0%), *Capillaria* spp. in two (1.3%), *Trichuris* spp. in two (1.3%), and liver fluke *(Fasciola* spp.) in one animal (0.7%) (Figure 2). Two or more parasite species was detected in six animals (3.9%).

### 3.3. GI Parasite Prevalence by Study Area

GI parasite positivity rates showed a progressive increase from Thong Pha Phum (14/49; 28.6%) through Sangkhla Buri (25/66; 37.9%) to the cross-border cattle with the Myanmar border (16/38; 42.1%) (Table 2).

Thong Pha Phum district (*n =* 49): Fourteen of 49 cattle (28.6%) were GI parasite positive. Strongyle-type nematode eggs were the only parasite species detected (14/49; 28.6%). No other parasite species were identified in this district, suggesting a limited parasite diversity compared with the other two study sites (Table 2).

Sangkhla Buri district (*n* = 66): Twenty-five of 66 cattle (37.9%) were GI parasite positive. Sangkhla exhibited the greatest parasite species diversity, with the following five species identified: Strongyle-type nematode eggs (14/66; 21.2%), rumen fluke (6/66; 9.1%), *Moniezia* spp. (3/66; 4.5%), *Capillaria* spp. (2/66; 3.0%), and *Trichuris* spp. (2/66; 3.0%). Co-infection with two or more species was recorded in two animals (3.0%) (Table 2).

Cross-border cattle (*n* = 38): Sixteen of 38 cattle (42.1%) were GI parasite positive, the highest area-level prevalence in this study. Strongyle-type nematode eggs were most common (12/38; 31.6%), followed by rumen fluke (7/38; 18.4%)—the highest rumen fluke prevalence across all three areas. Liver fluke was detected in one animal (2.6%) and was identified exclusively at this site, possibly reflecting ecological conditions at the Myanmar border that favor snail intermediate hosts. Two or more parasite species were most frequent at this site, occurring in four animals (10.5%) (Table 2).

### 3.4. Risk Factor Analysis for Gastrointestinal Parasite Infection

Univariable analysis was evaluated to assess associations between host-level and farm management variables and overall GI parasite positivity, using Chi-squared tests or Fisher’s exact test, as appropriate (Table 3). None of the sixteen variables examined was significantly associated with GI parasite positivity (all *p* > 0.05; Fisher’s exact test used where any expected cell count was below five: age, anti-parasitic treatment, cat roaming, and abortion history), and all 95% confidence intervals for the odds ratios were wide and crossed one, reflecting the sample size available for subgroup comparisons. Only univariable, one-variable-at-a-time testing was performed; a multivariable model was not fitted and could plausibly alter this picture by accounting for the collinearity between location, sex, and breed described in Section 3.4.1.

#### 3.4.1. Host-Level Factors

Male cattle (43.1%; 28/65) and local-breed cattle (40.0%; 38/95) showed a numerically higher, but not statistically significant, prevalence than females (30.7%; 27/88) and crossbred cattle (29.3%; 17/58), respectively (OR = 1.71, 95% CI 0.88–3.33, *p* = 0.159 for sex; OR = 1.61, 95% CI 0.80–3.23, *p* = 0.245 for breed) (Table 3); both trends are addressed as potential confounding by location in the Discussion. Abortion history was recorded in only three animals, precluding meaningful interpretation of its association with GI parasite status (OR = 3.66, 95% CI 0.32–41.32, *p* = 0.293, Fisher’s exact test). Age, dichotomized at 1 year, was not significantly associated with GI parasite positivity: young cattle (44.4%; 4/9) and adult cattle (35.4%; 51/144) did not differ significantly (OR = 0.69, 95% CI 0.18–2.67, *p* = 0.723, Fisher’s exact test), though the small number of young animals limits this comparison. Pregnancy status was similarly not associated with infection: pregnant animals (35.7%; 10/28) and non-pregnant animals (36.0%; 45/125) showed almost identical positivity (OR = 0.99, 95% CI 0.42–2.32, *p* = 1.000) (Table 3).

#### 3.4.2. Farm Environment and Management Factors

None of the farm-level variables tested—water source (OR = 1.09, 95% CI 0.54–2.20, *p* = 0.945), anti-parasitic treatment (OR = 0.83, 95% CI 0.22–3.08, *p* = 0.747, Fisher’s exact test), anti-ectoparasitic treatment (OR = 0.45, 95% CI 0.16–1.26, *p* = 0.200), ectoparasite infestation (OR = 0.77, 95% CI 0.38–1.55, *p* = 0.584), or the presence of dogs, cats, chickens, wild birds, or rats—were significantly associated with GI parasite positivity (Table 3). The strongest, though still non-significant, trend was a lower infection odd among cattle with cats present on the farm (OR = 0.56, 95% CI 0.29–1.10, *p* = 0.126).

## 4. Discussion

This survey extends earlier parasitological work in Kanchanaburi Province, where Income et al. [15] reported a GI helminth prevalence of 35.7% in 157 cattle examined by microscopic fecal examination. The overall prevalence recorded here was closely comparable, confirming the persistent burden of GI parasitism in this province. Direct comparison with prevalence figures reported elsewhere, however, needs to account for substantial differences in agroecology, farming system, and study design rather than percentage values alone. Thanasuwan et al. [4] documented 52.3% in Kalasin Province on the drier northeastern where cattle are typically kept under more intensive, paddock-based smallholder systems, and Suchawan et al. [7] recorded over 60% in Sa Kaeo Province, another eastern border province with monsoon-forest terrain and, like Kanchanaburi, active cross-border livestock movement, while Kaewthamasorn and Wongsamee [14] reported 45.3% in Nan Province with peak infections during the rainy season. Across the border, Soe et al. [20] recorded a substantially higher prevalence of 79.5% in cattle of the Ayeyarwady Division, Myanmar—a low-lying river-delta region with extensive irrigation and near-permanent pasture moisture that is ecologically more favorable for larval survival than the drier, hillier terrain sampled here, and which is relevant because the border-site cattle in the present study were likely sourced from or traded through Myanmar. Beyond these agroecological contrasts, the comparatively lower prevalence recorded here may reflect the dry-season sampling window (December 2022–January 2023), which limits larval survival on pasture [9], together with the high reported anthelmintic treatment coverage (93.5%) in this population; the prevalence and diversity contrasts discussed below should therefore be read as indicative of differing ecological and management contexts rather than as a direct ranking of parasite burden.

Strongyle-type nematodes were the most prevalent group, consistent with their established dominance in tropical ruminants and with the elevated Strongyle-type egg prevalence reported in fighting bulls in southern Thailand [21], and with Income et al.’s [15] earlier identification of *Cooperia* spp., *Haemonchus* spp., *Oesophagostomum* spp., and *Trichostrongylus* spp. as the predominant GI parasites in this same province. Notably, Strongyle-type infection was detected even in Thong Pha Phum, where all sampled cattle had received anthelmintic treatment; this finding alone cannot confirm reduced anthelmintic efficacy or resistance, since treatment timing, dosage, and FECRT data were not available in this study, but it is consistent with a broader pattern reported in a meta-analysis of Indonesian cattle, where Strongyle-type nematodes persisted as the most intractable GI parasites despite widespread anthelmintic use [22].

Rumen fluke and liver fluke followed a distribution consistent with reliance on river water: both were detected only at the border and Sangkhla sites, where cattle drank predominantly from the Khwae Noi River system, and were absent in Thong Pha Phum, where piped water was used. This pattern aligns with previous reports of *Paramphistomum* spp. in river-adjacent cattle populations in Thailand [23] and Myanmar [20], and reflects the dependence of both flukes on aquatic snail intermediate hosts that thrive in slow-moving freshwater. Even though *Fasciola* spp. was found only in a single animal, this is an indication that the parasite occurs in the study area, meaning there is an environment conducive to its survival. There is a need for more investigations on the prevalence of this parasite through molecular techniques with a larger sample size [24,25,26].

*Moniezia* spp. showed a more restricted distribution, detected only in Sangkhla at a low prevalence (3/66, 4.5%), consistent with the generally limited pathogenicity of this tapeworm in adult cattle [27]. Its transmission depends on oribatid mites as obligate intermediate hosts, which acquire *Moniezia* eggs from pasture and soil and are in turn ingested by grazing cattle; this pasture- and soil-mite-dependent cycle is mechanistically distinct from the water-dependent trematodes discussed above, which may explain why its distribution did not track the river-water gradient. A recent global meta-analysis estimated a pooled *Moniezia expansa* prevalence of 21.3%, well above the 2.0% recorded here, suggesting either a lower oribatid mite density in Kanchanaburi’s pastures or a sampling window that under-captured seasonal tapeworm shedding [27].

The border site recorded the highest overall prevalence, the highest parasitic diversity rate, and the only liver fluke detections, making it epidemiologically the most complex of the three sites. All border cattle were exclusively male, suggesting these animals were in transit for trade rather than resident—consistent with regional export data showing over 12,000 head of cattle officially moved from Myanmar into Thailand in 2021–2022 [28]. Itinerant herds crossing ecological zones without adequate treatment breaks are expected to accumulate multiple infections, which plausibly explains the elevated prevalence rate at this site relative to the more settled herds in Thong Pha Phum and Sangkhla, and is consistent with *Fasciola* spp. being a parasite commonly introduced into resident herds through cross-border livestock trade [24].

None of the individual risk factors reached statistical significance in univariable analysis (including area itself, *p* = 0.389), most plausibly reflecting structural rather than biological limits of this study: the sample size, while adequate for prevalence estimation, was underpowered for detecting moderate odds ratios in small subgroups, and extensive collinearity at the border site—where all animals were simultaneously male, local-breed, river-water-dependent, and from the highest-prevalence district—makes it statistically impossible to isolate any single factor’s contribution from that of location itself. The non-significant trends toward higher prevalence in male and local-breed cattle (OR = 1.71 and 1.61, respectively) are therefore best interpreted as confounding by site rather than independent effects, consistent with Suchawan et al.’s [7] similar finding after adjusting for site. Reservoir-animal presence (dogs, cats, and rats) also showed no association, likely because these animals were present on almost every farm, leaving little variation to detect an effect—in contrast to Rerkyusuke et al.’s [11] finding of an association in a more heterogeneous goat population. Multivariable or mixed-effect models with farm as a random effect would better disentangle these confounded factors in future work.

Several taxa identified here also carry zoonotic potential relevant to a One Health perspective. *Trichostrongylus* spp. has been linked to human trichostrongyliasis in northeast Thailand and Lao PDR [29], and *Capillaria* spp., *Trichuris* spp., and *Fasciola* spp. include species with documented capacity to infect humans via the fecal-oral or aquatic-vegetation route [9,24,30]. Given that cattle entering Thailand from Myanmar carry a substantially higher parasite burden [20], and that communities along this border share the same river systems with their livestock [24], compulsory fecal examination and quarantine before mixing with resident herds—supported by iEPGnter-country veterinary coordination and consistent with existing cross-border disease surveillance models in the Mekong region [8,31]—would be a logical control measure to limit the introduction of novel parasite taxa into the resident Thai cattle population [32].

Beyond their epidemiological description, the parasites identified here carry economic and ecological significance that merits brief consideration. Even subclinical GI parasitism of the kind documented here is associated with reduced weight gain, milk yield, and reproductive performance in cattle [33], and gastrointestinal nematode and trematode infections are estimated to cost beef and dairy producers worldwide billions of US dollars annually [34], costs that are rarely quantified in smallholder cross-border settings such as this one but are unlikely to be negligible given the 35.9% overall positivity recorded here. Ecologically, the persistence of trophically transmitted taxa such as *Paramphistomum* spp., *Fasciola* spp., and *Moniezia* spp. in this landscape is not only a direct infection risk but also an indicator of the continued presence of their obligate intermediate hosts—freshwater snails for both flukes and oribatid mites for *Moniezia*—within the farm environment, reflecting broader ecological suitability for these host groups that sustains transmission to any grazing or browsing animal sharing the same pasture and water sources, independent of cattle management practices alone.

Several limitations should be acknowledged. First, eggs per gram (EPGs) were not quantified, so infection intensity could not be assessed, and results are limited to presence/absence data. A quantitative technique such as the McMaster method is recommended in future surveys. Second, the three study populations differed considerably in size (*n* = 49, 66, and 38) and were sampled by convenience rather than random selection; this imbalance, together with the near-complete collinearity between the border site and cattle sex, breed, and water source, likely limited statistical power to detect genuine risk-factor associations and limited the feasibility of fitting a robust multivariable or mixed-effect model. Therefore, the univariable associations reported here should be interpreted as exploratory rather than adjusted estimates. Samples were also collected from a limited number of farms rather than independently sampled animals, so farm-level clustering was not accounted for and may have resulted in underestimated standard errors. Third, sampling was restricted to a single dry-season window, so seasonal variation in prevalence and seasonal diversity was not captured. Finally, parasite identification relied on egg morphology under light microscopy, without larval culture or molecular confirmation; genus-level findings, including inferences regarding zoonotic potential, species diversity, and parasite transmission pathways, should therefore be interpreted with caution. Farm-level animal-presence variables (dogs, cats, chickens, wild birds, and rats) were owner-reported and cross-checked only at a single visit, so recall or reporting bias cannot be excluded. Lastly, egg detection in treated animals should not be read as evidence of anthelmintic resistance without fecal egg count reduction testing (FECRT) and verified treatment records, also without knowing the drug type and dosage, which future work should incorporate.

## 5. Conclusions

Gastrointestinal parasite infection remains prevalent (35.9%) among cattle in Kanchanaburi province despite high anthelmintic coverage, and it increases toward the Thai–Myanmar border, where cross-border cattle movement and shared river-water use converge to create the greatest parasite diversity, including the only detections of *Fasciola* spp. This border region therefore represents both a One Health concern, given the zoonotic potential of several taxa identified, and a priority target for cross-border quarantine screening and coordinated veterinary surveillance. The persistence of infection despite reported anthelmintic treatment, while not confirmatory of resistance in the absence of FECRT data, supports the value of targeted, egg-count-guided deworming protocols and periodic FECRT monitoring rather than reliance on blanket treatment alone. To our knowledge, this is the first study to directly compare local and cross-border cattle populations within Kanchanaburi province, providing a baseline against which future seasonal, molecular, and multivariable studies of GI parasite epidemiology in this border region can build.

## Figures and Tables

**Table 1:** Demographic and farm management characteristics of sampled cattle by study area, Kanchanaburi province, 2022.

Characteristic		Thong Pha Phum (*n* = 49)	Sangkhla Buri (*n* = 66)	Cross-Border Cattle (*n* = 38)	Total (*n* = 153)
Sex	Female	36 (73.5%)	52 (78.8%)	0 (0.0%)	88 (57.5%)
	Male	13 (26.5%)	14 (21.2%)	38 (100.0%)	65 (42.5%)
Age	Young	6 (12.2%)	3 (4.5%)	0 (0.0%)	9 (5.9%)
	Adult	43 (87.8%)	63 (95.5%)	38 (100.0%)	144 (94.1%)
Breed	Local breed	9 (18.4%)	48 (72.7%)	38 (100.0%)	95 (62.1%)
	Crossbred (Mix AB)	40 (81.6%)	18 (27.3%)	0 (0.0%)	58 (37.9%)
Pregnancy status	Non-pregnant	30 (61.2%)	57 (86.4%)	38 (100.0%)	125 (81.7%)
	Pregnant	19 (38.8%)	9 (13.6%)	0 (0.0%)	28 (18.3%)
Water source	River	12 (24.5%)	51 (77.3%)	38 (100.0%)	101 (66.0%)
	Piped	37 (75.5%)	15 (22.7%)	0 (0.0%)	52 (34.0%)
Animals on a farm	Dog present	44 (89.8%)	51 (77.3%)	38 (100.0%)	133 (86.9%)
	Dog roaming	44 (89.8%)	44 (66.7%)	38 (100.0%)	126 (82.4%)
	Cat present	37 (75.5%)	41 (62.1%)	0 (0.0%)	78 (51.0%)
	Cat roaming	0 (0.0%)	8 (12.1%)	0 (0.0%)	8 (5.2%)
	Chicken present	37 (75.5%)	14 (21.2%)	38 (100.0%)	89 (58.2%)
	Wild bird present	49 (100.0%)	0 (0.0%)	0 (0.0%)	49 (32.0%)
	Rat present	49 (100.0%)	45 (68.2%)	38 (100.0%)	132 (86.3%)
Clinical findings	Ectoparasite infestation	47 (95.9%)	56 (84.8%)	0 (0.0%)	103 (67.3%)
	Abortion history	2 (4.1%)	1 (1.5%)	0 (0.0%)	3 (2.0%)
Antiparasitic treatment	Anti-parasitic (internal)	49 (100.0%)	56 (84.8%)	38 (100.0%)	143 (93.5%)
	Anti-ectoparasitic	49 (100.0%)	49 (74.2%)	38 (100.0%)	136 (88.9%)

Values are presented as a number (percentage). Mix AB = crossbred cattle, referring to crosses between Bos indicus and Bos taurus breeds the predominant crossbreeding system in Thai cattle production.

**Table 2:** Prevalence of gastrointestinal parasite species by study area, Kanchanaburi province, 2022.

Parasite Species	Thong Pha Phum *n* = 49, *n* (%)	Sangkhla Buri *n* = 66, *n* (%)	Cross-Border Cattle *n* = 38, *n* (%)	Total *n* = 153, *n* (%)
Strongyle-type nematode	14 (28.6%)	14 (21.2%)	12 (31.6%)	40 (26.1%)
*Paramphistomum* spp.	0 (0.0%)	6 (9.1%)	7 (18.4%)	13 (8.5%)
*Moniezia* spp.	0 (0.0%)	3 (4.5%)	0 (0.0%)	3 (2.0%)
*Capillaria* spp.	0 (0.0%)	2 (3.0%)	0 (0.0%)	2 (1.3%)
*Trichuris* spp.	0 (0.0%)	2 (3.0%)	0 (0.0%)	2 (1.3%)
*Fasciola* spp.	0 (0.0%)	0 (0.0%)	1 (2.6%)	1 (0.7%)
Overall, GI parasite positive	14 (28.6%)	25 (37.9%)	16 (42.1%)	55 (35.9%)
≥2 species	0 (0.0%)	2 (3.0%)	4 (10.5%)	6 (3.9%)

Values are presented as a number (percentage).

**Table 3:** Univariable risk factor analysis for gastrointestinal parasite infection in cattle, Kanchanaburi province, 2022.

Variable	Category	Positive/Total	Prevalence (%)	OR	95% CI	*p*-Value
Sex	Female (ref)	27/88	30.7%	1.00	—	—
	Male	28/65	43.1%	1.71	0.88–3.33	0.159
Age	Young (ref)	4/9	44.4%	1.00	—	—
	Adult	51/144	35.4%	0.69	0.18–2.67	0.723 *
Breed	Crossbred (ref)	17/58	29.3%	1.00	—	—
	Local breed	38/95	40.0%	1.61	0.80–3.23	0.245
Pregnancy status	Non-pregnant (ref)	45/125	36.0%	1.00	—	—
	Pregnant	10/28	35.7%	0.99	0.42–2.32	1.000
Water source	Piped (ref)	18/52	34.6%	1.00	—	—
	River	37/101	36.6%	1.09	0.54–2.20	0.945
Anti-parasitic treatment	No (ref)	4/10	40.0%	1.00	—	—
	Yes	51/143	35.7%	0.83	0.22–3.08	0.747 *
Anti-ectoparasitic treatment	No (ref)	9/17	52.9%	1.00	—	—
	Yes	46/136	33.8%	0.45	0.16–1.26	0.200
Ectoparasite infestation	Absent (ref)	20/50	40.0%	1.00	—	—
	Present	35/103	34.0%	0.77	0.38–1.55	0.584
Dog in farm	No (ref)	9/20	45.0%	1.00	—	—
	Yes	46/133	34.6%	0.65	0.25–1.67	0.512
Dog roaming	No (ref)	10/27	37.0%	1.00	—	—
	Yes	45/126	35.7%	0.94	0.40–2.24	1.000
Cat in farm	No (ref)	32/75	42.7%	1.00	—	—
	Yes	23/78	29.5%	0.56	0.29–1.10	0.126
Cat roaming	No (ref)	51/145	35.2%	1.00	—	—
	Yes	4/8	50.0%	1.84	0.44–7.68	0.459 *
Chicken in farm	No (ref)	22/64	34.4%	1.00	—	—
	Yes	33/89	37.1%	1.12	0.57–2.20	0.863
Wild bird presence	No (ref)	41/104	39.4%	1.00	—	—
	Yes	14/49	28.6%	0.61	0.29–1.28	0.261
Rat in the farm	No (ref)	6/21	28.6%	1.00	—	—
	Yes	49/132	37.1%	1.48	0.54–4.05	0.608
Abortion history	No (ref)	53/150	35.3%	1.00	—	—
	Yes	2/3	66.7%	3.66	0.32–41.32	0.293 *
Study area †	Thong Pha Phum (ref)	14/49	28.6%	1.00	—	—
	Sangkhla	25/66	37.9%	—	—	0.389 †
	Cross-border Cattle	16/38	42.1%	—	—	0.389 †

OR = odds ratio; CI= confidence interval; ref = reference category; — = not applicable. The chi-square test was used, except when any cell expectation was <5, in which case Fisher’s exact test was conducted (*). † Chi-square test across all three area categories simultaneously (df- = 2); individual pairwise Ors and CIs were not estimated. Statistical significance threshold: *p* < 0.05.

## Data Availability

The original contributions presented in this study are included in the article. Further inquiries can be directed to the corresponding author.

## Abbreviations

The following abbreviations are used in this manuscript:

OR	Odd Ratio
CI	Confidence Interval
EPG	Egg Per Gram
FECRT	Fecal Egg Count Reduction Test
Df	Degree of Freedom
Lao PDR	Lao People’s Democratic Republic
NRCT	National Research Council of Thailand
